# Mapping PedsQL™ scores to CHU9D utility weights for children with chronic conditions in a multi-ethnic and deprived metropolitan population

**DOI:** 10.1007/s11136-023-03359-4

**Published:** 2023-02-23

**Authors:** Clare B. Kelly, Marina Soley-Bori, Raghu Lingam, Julia Forman, Lizzie Cecil, James Newham, Ingrid Wolfe, Julia Fox-Rushby

**Affiliations:** 1 Institute for Women and Children’s Health, King’s Health Partners, London, UK; 2 Department of Population Health, School of Life Course and Population Sciences, King’s College London, London, UK; 3 School of Women’s and Children’s Health, University of New South Wales, Sydney, Australia; 4 Department of Women’s and Children’s Health, School of Life Course and Population Sciences, King’s College London, London, UK; 5 Faculty of Health and Life Sciences, Northumbria University, Newcastle upon Tyne, UK; 6 NIHR Biomedical Research Centre, Guy’s and St Thomas’ NHS Foundation Trust and King’s College London, London, UK; 7 Faculty of Life Sciences and Medicine, School of Life Course & Population Sciences, King’s College London, Guy’s Campus, Great Maze Pond, London, SE1 1UL UK

**Keywords:** Health-related quality of life, Patient reported outcomes, Mapping algorithms, Children and young people

## Abstract

**Purpose:**

The Child Health Utility-9 Dimensions (CHU9D) is a patient-reported outcome measure to generate Quality-Adjusted Life Years (QALYs), recommended for economic evaluations of interventions to inform funding decisions. When the CHU9D is not available, mapping algorithms offer an opportunity to convert other paediatric instruments, such as the Paediatric Quality of Life Inventory™ (PedsQL), onto the CHU9D scores.

This study aims to validate current PedsQL to CHU9D mappings in a sample of children and young people of a wide age range (0 to 16 years of age) and with chronic conditions. New algorithms with improved predictive accuracy are also developed.

**Methods:**

Data from the Children and Young People’s Health Partnership (CYPHP) were used (*N* = 1735). Four regression models were estimated: ordinal least squared, generalized linear model, beta-binomial and censored least absolute deviations. Standard goodness of fit measures were used for validation and to assess new algorithms.

**Results:**

While previous algorithms perform well, performance can be enhanced. OLS was the best estimation method for the final equations at the total, dimension and item PedsQL scores levels. The CYPHP mapping algorithms include age as an important predictor and more non-linear terms compared with previous work.

**Conclusion:**

The new CYPHP mappings are particularly relevant for samples with children and young people with chronic conditions living in deprived and urban settings. Further validation in an external sample is required.

**Trial registration number** NCT03461848; pre-results.

**Supplementary Information:**

The online version contains supplementary material available at 10.1007/s11136-023-03359-4.

## Introduction

Health-related quality of life (HRQoL) is seen as an increasingly important outcome in clinical, epidemiological, and health economics studies in children and young people [1, 2]. In comparison to morbidity and mortality measures, HRQoL instruments—such as the Paediatric Quality of Life Inventory™ (PedsQL) and the Child Health Utility-9 Dimensions (CHU9D)—provide a comprehensive, patient-centric assessment of the impact of illness on a child’s health [3, 4]. These tools support the increasingly recognised role of children and young people as independent patients whose perceptions of disease and opinions ought to be understood [5, 6]. HRQoL measures are recommended by the National Institute for Health Care Excellence (NICE) in cost-effectiveness analyses of interventions to inform funding decisions [7]. Specifically for children and young people, NICE guidance favours the use of generic measures with good psychometric performance in the relevant age range [8].

The PedsQL is a generic non-preference-based instrument for use in children and adolescents [9]. Six age-specific versions (0–12 months, 13–24 months, 2–4 years, 5–7 years, 8–12 years, and 13–17 years) ensure validated questions are developmentally appropriate. Its 23 to 45 items measure aspects of physical, emotional, and social health and wellbeing, along with physical symptoms, cognitive functioning, and school functioning. This instrument distinguishes between healthy children and those with acute and chronic conditions, as well as across severity levels within these conditions [10]. The CHU9D, a paediatric generic preference-based instrument with weights obtained from a sample of the UK adult general population, has both the ability to ‘measure’ and ‘value’ health status [11, 12]. Based on nine items measuring mental and physical health, schoolwork, and social activities, a health state is generated, with weights reflecting population preferences that produce utility values on a 0 to 1 scale. These utility weights can be attached to lengths of life to calculate Quality-Adjusted Life Years, QALYs [13]. A cost-utility analysis with QALYs is part of the recommended economic evaluation methodology by NICE [14], making measures such as the CHU9D essential when considering health effects of paediatric interventions. Both the PedsQL and the CHU9D have been extensively used in a variety of paediatric populations such as children with asthma, mental health issues, and eczema [10, 15].

Preference-based measures such as the CHU9D tend to be less often used in clinical studies [16, 17]. When the CHU9D instrument is not available, QALYs can be estimated from the PedsQL responses using existing mapping algorithms [18–20]. This approach is also referred to as “transfer to utility regression” [21] and often requires not only PedsQL scores but also demographic characteristics of the study sample. Aside from economic evaluation applications in decision making, the ability to estimate CHU9D from PedsQL scores can be useful when summarising existing evidence into a single HRQoL scale. Mapping algorithms also offer the opportunity of reducing respondent burden and administration costs. However, the practicality of these algorithms may be limited by their predictive ability and generalisability to the age and disease prevalence in the target population [22, 23].

A methodological challenge of the mapping algorithms is to ensure consistency in their predictive accuracy across population subgroups. Existing algorithms have been developed using populations with limited age ranges and medical conditions [18–20]. Mpundu-Kaamdma and colleagues developed mapping algorithms between PedsQL and CHU9D based on 755 Australian adolescents aged 15–17 years [18]. Lambe and colleagues used data on 563 children in the UK, aged between 5 and 13 years, taking part in a randomised controlled trial for treatment of corticosteroid-sensitive nephrotic syndrome [20]. The most recent mapping study by Sweeney et al. assessed the predictive accuracy of the Mpundu-Kaamdma and Lambe algorithms using a cohort of 1801 Australian children, aged 10–12 years [18] (external validation), and developed new mappings (internal validation). This study led to improved total, dimension, and item-level mappings being developed with better predictive accuracy. The authors indicated that further external validation of the ‘new’ mappings was needed given the narrow age band and that the performance of the algorithms among children with relatively poor HRQoL was worse than the full sample. Poor performance accuracy of existing mappings among children with medical conditions was also noted by Mpundu-Kaamdma [24].

We aim to externally validate the most recent PedsQL to CHU9D mappings for a sample of children and young people of a wide age range (0 to 16 years of age) and with chronic conditions, from an ethnically diverse and deprived area in South London. In addition, we assess whether new algorithms could improve the predictive accuracy for this unique population.

## Methods

### Design and participants

This study used baseline data from a cluster randomised controlled trial designed to evaluate the impact of the Children and Young People’s Health Partnership (CYPHP) Evelina London Model of Care, an innovative approach to integrated healthcare delivery. The CYPHP study design and intervention components are outlined in detail in the published trial protocol paper [25]. The trial population included children and young people under 16 years of age registered to a general practice in Southwark or Lambeth. To assess the impact of CYPHP on patient-reported outcomes, a subset of the trial sample with specific conditions (asthma, eczema, or constipation) were further consented, and self-reported questionnaires administered, including both the PedsQL and the CHU9D. For assessing the performance of existing mappings in the CYPHP sample (external validation) and mapping development (internal validation) purposes, we selected study participants in either arm of the trial who completed or had enough responses to generate final scores for both baseline questionnaires (including the appropriate PedsQL age version), from April 2018 (start of CYPHP) to February 2021 (end of baseline recruitment) (*n* = 1735). We used the resulting sample for the external validation and randomly divided the study sample into estimation (80%) and validation (20%) groups for the exploration of new algorithms [17].

### HRQoL measures

The PedsQL was developed from a cohort of paediatric cancer patients 8–18 years of age [9]. The most recent version (PedsQL 4.0), resulted from several enhancements of the initial questionnaire, including increased item pool to ensure coverage of the core WHO health dimensions [26], expansion of appropriate age range (2–18 years), and more sensitive scaling range [27]. Both self-completed and parent-proxy versions are available. The total number of questions ranges from 23 to 45, which can be grouped into four dimensions for children aged two or above (physical, emotional, social, and school functioning) or five dimensions for children under the age of two (physical symptoms, and physical, emotional, social and cognitive functioning). Item scaling is on a 5-point Likert scale from 0 (never) to 4 (almost always), with a recall period of last month. Missing values are replaced by the mean of completed items in the scale. If more than half of the items within a dimension are missing, the final score is not generated. Final scores are transformed into a 0–100 scale, with higher values indicating better HRQoL [28].

The CHU9D was developed based on in-depth qualitative interviews with children 7–11 years of age with a variety of chronic and acute health problems [11, 29]. The instrument was then validated among younger children (5–7 years) [30, 31] and adolescent (11–17 years of age) populations [32]. The questionnaire is self-completed, with proxy completion available for younger children. Five response options (indicating increasing levels of severity) are provided for each of the nine questions, with a recall period of today or last night. No missing values are allowed for calculating the final CHU9D score. Population preference weights—obtained based on the standard gamble method—are applied to the health status defined by the nine responses to generate utility values in the 0–1 scale corresponding to QALYs, where 0 indicates death and 1 perfect health [13].

### Statistical analysis

Sociodemographic characteristics (age, sex, and index of multiple deprivation) and medical conditions of participants in the study were summarized using means and standard deviations for continuous variables and counts and percentages for categorical variables. The relationship between the two instruments was analysed using Spearman correlation coefficient and presented through scatter plots.

#### External validation of previously published mapping algorithms

We externally validated three mapping algorithms developed by Sweeney et al. [19], based on item, summary, and total PedsQL scores. These mappings are estimated regression equations with the CHU9D as the dependent variable and the PedsQL as the main independent variable. PedsQL squared terms are included to allow for a non-linear relationship between the two variables. Models were also adjusted for age and sex based on previous model specifications. The first step of the external validation was to apply each of the individual mapping algorithms to the PedsQL scores (including age and sex respectively) and the CHU9D scores. The resulting CHU9D predicted scores were then compared to the CHU9D observed scores and goodness of fit measures calculated to evaluate the accuracy of the estimated CHU9D scores for our study sample.

#### Developing new mapping algorithms

Using the CYPHP cohort, we investigated whether we could improve the predictive accuracy of the mapping algorithms. Our full study sample was divided into estimation (80%) and validation (20%) samples, using random sampling without replacement. We considered the following model specifications for the total, dimension, and item scores based on previous mapping development papers [18–20]:

1 $$\mathrm{CHU}9\mathrm{D}=\alpha +{\beta }_{1}\mathrm{PedsQL}+{\beta }_{2}{\mathrm{PedsQL}}^{2}+{\gamma }_{1}\mathrm{Age}+{\gamma }_{2}\mathrm{Sex},$$

2 $$\mathrm{CHU}9\mathrm{D}=\alpha +{\sum_{i=1}^{I}\beta }_{i}{\mathrm{PedsQL}}_{\mathrm{DIM}}+{\sum_{k=1}^{K}\beta }_{k}\mathrm{PedsQ}{{\mathrm{L}}_{\mathrm{DIM}}}^{2}+{\gamma }_{1}\mathrm{Age}+{\gamma }_{2}\mathrm{Sex},$$

3 $$\mathrm{CHU}9\mathrm{D}=\alpha +{\sum_{j=1}^{J}\beta }_{j}{\mathrm{PedsQL}}_{\mathrm{ITEM}}+{\sum_{l=1}^{L}\beta }_{l}\mathrm{PedsQ}{{\mathrm{L}}_{\mathrm{ITEM}}}^{2}+{\gamma }_{1}\mathrm{Age}+{\gamma }_{2}\mathrm{Sex},$$

where $\alpha$ is a constant, $\mathrm{PedsQL}$ is overall total PedsQL score, ${\mathrm{PedsQL}}_{\mathrm{DIM}}$ corresponds to the PedsQL dimension scores, ${\mathrm{PedsQL}}_{\mathrm{ITEM}}$ corresponds to the PedsQL item scores, and ${\beta }_{i}$, ${\beta }_{k}$, ${\beta }_{j}, {\beta }_{l}$,${\gamma }_{1}$, and ${\gamma }_{2}$ are parameter estimates. Subscripts *i* and *k* range from 4 to 5 dimensions, while subscripts *j* and *l* from 23 to 45 items, dependent on the age versions of the PedsQL questionnaire. The variable ‘Sex’ is defined as 0—female and 1—male.

Stepwise variable selection using the OLS estimator and based on Akaike Information Criterion (AIC) was used to identify the final specification for each of the three equations [17, 33, 34]. As a sensitivity analysis, forward selection with an entry variable criterion of *p*-value ≤ 0.1 was employed and compared to the AIC-based approach through goodness of fit measures. Each final equation was estimated using four different functional forms: ordinary least squares regression (OLS), generalised linear model with the negative binomial distribution and the logit link (GLM) to accommodate the skewness and heteroscedasticity in the estimation sample, BETA regression to account for non-linear functional forms of the predictors, and censored least absolute deviations estimator (CLAD) to consider heteroscedasticity and outliers. The presence of multicollinearity among selected regressors was assessed via the Variance Inflating Factor (VIF).

#### Goodness of fit measures

We calculated a series of goodness of fit measures to assess the performance of the mapping algorithms. For the external validation, these indicators were computed based on the entire sample, while for the internal validation only the validation sample was used. The correlation coefficient, plots of predicted versus observed values, and the *R*^2^ were presented to understand the strength of the relationship between predicted and observed values and the proportion of explained variance by model regressors. To assess predictive accuracy, mean absolute errors (MAE), mean squared errors (MSE), and root mean squared error (RMSE) were computed, with lower values indicating better accuracy. MSE is more sensitive to outliers than MAE because the squared, rather than the absolute function, is applied to residuals [35]. Mean, minimum and maximum values of observed and predicted CHU9D scores were also compared, along with the average error and the percentage of observations with an absolute error smaller than 0.05. We defined the ‘best’ mapping algorithm as that which performed the best across the majority of these statistics. Goodness of fit statistics were computed for the overall sample and also by age (0–12 months, 13–24 months, 2–4 years, 5–7 years, 8–12 years, 13–17 years) and medical conditions (asthma, eczema, constipation, multimorbidity) subgroups.

Linear regression analysis was carried out, using the validation dataset, to determine the relationship between the results of the “best” performing algorithms using the CYPHP cohort and the mappings previously defined by Sweeney et al. Finally, goodness of fit measures were computed for the CYPHP “best” performing algorithm, with and without including selected demographic variables to better understand their explanatory power.

SAS version 9.4, IBM SPSS Statistics 27 and STATA v16 (StataCorp LP, College Station, Texas, USA) were used for analyses. We followed the Professional Society for Health Economics and Outcomes Research (ISPOR) good practice recommendations for mapping health-state utility from non-preference-based outcome measures [17].

#### Ethical considerations

The data are derived from the CYPHP study, which received ethical approval from South West-Cornwall and Plymouth Research Ethics Committee (REC Reference: 17/SW/0275).

## Results

### Sample and descriptive data

**Fig. 1:**
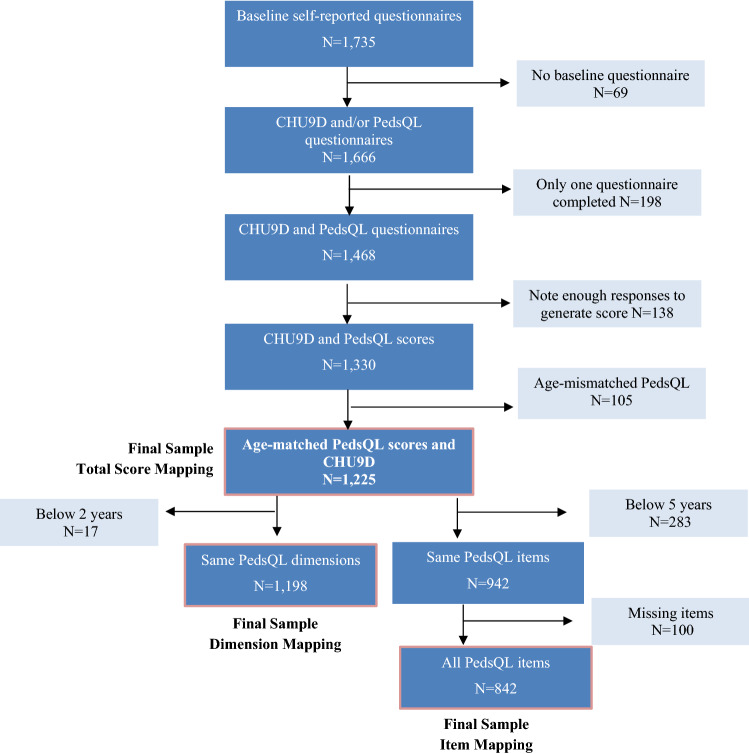
Study sample inclusion section flow

A total of 1735 individuals completed baseline self-reported questionnaires. For total score mapping purposes, this sample was reduced to 1225 after excluding individuals due to: not having completed both baseline PedsQL and CHU9D questionnaires; insufficient responses to generate total scores; age-mismatched PedsQL questionnaires; or missing sex (Fig. 1). Further exclusion criteria were needed to select the dimension and item-level mapping samples. Total scores are generated in all age-specific PedsQL questionnaires, but score calculation for dimensions and items do vary across versions. Children under 2 years of age were excluded for the dimension score mapping, resulting in a final sample of 1198 individuals. Responses from children below 5 years of age were disregarded for the item-level mapping due to heterogeneity in item content, leaving 842 individuals for analysis.

**Fig. 2:**
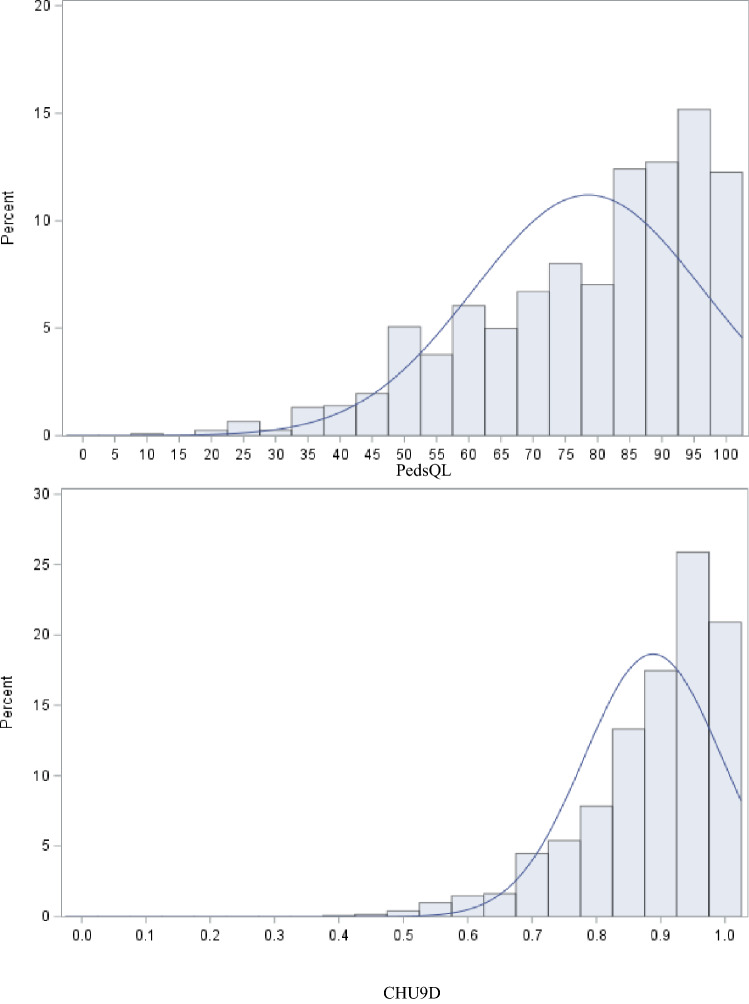
Distribution of the total PedsQL and CHU9D scores, full sample. *Notes N* = 1225. Mean PedsQL was 78.57 (17.82), mean CHU9D was 0.888 (SD = 0.11)

**Fig. 3:**
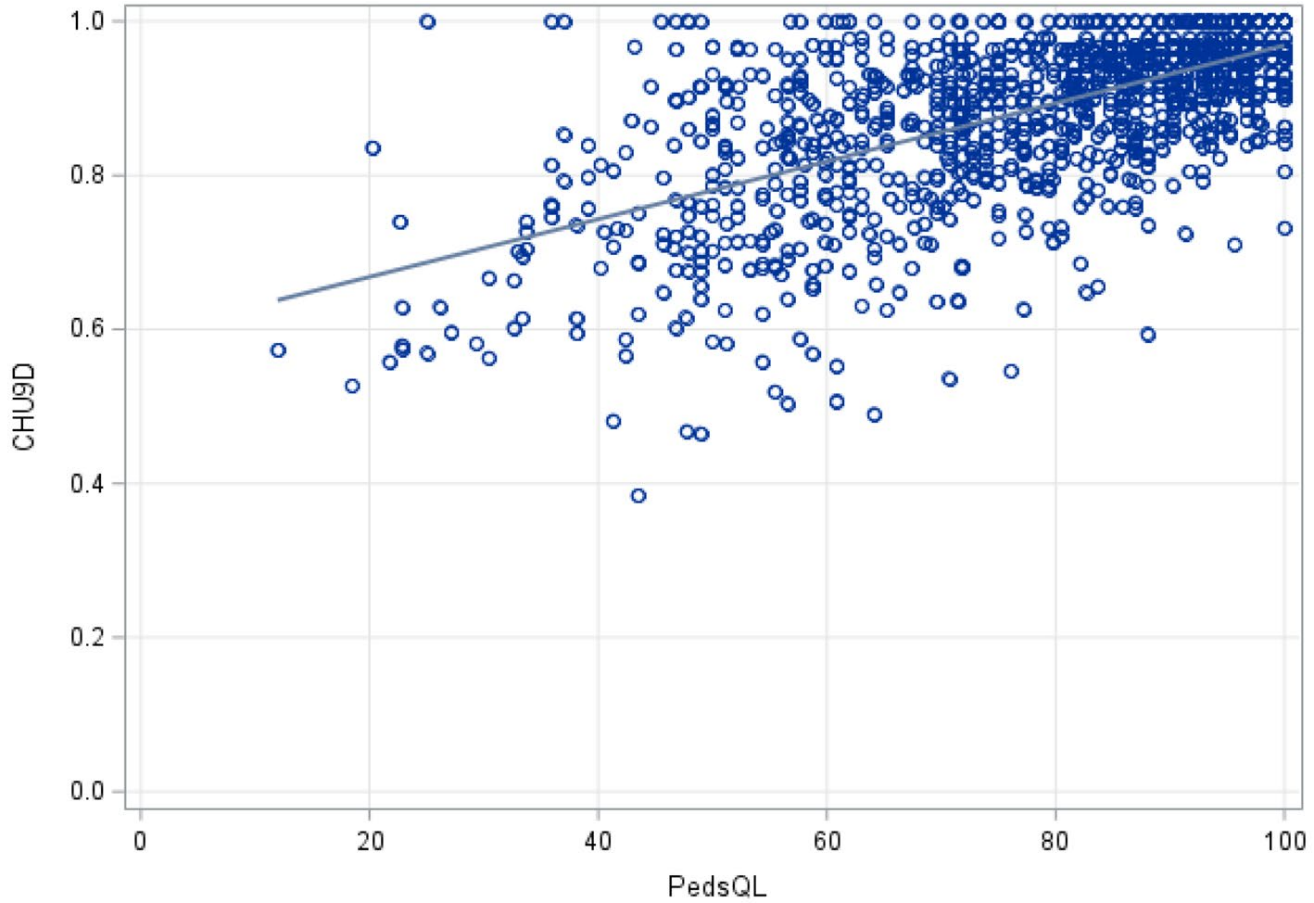
Scatter plot displaying total PedsQL and CHU9D scores. *Notes N* = 1225. Pearson correlation coefficient = 0.625 (*p* < 0.0001)

In the full sample, 53% were males (Table 1). The overall mean age of participants was 8.07 years (SD = 4.08), with 2.2% of the sample below 24 months of age, 27.2% between 2 and 4 years, 20.6% between 5 and 7 years, 33.7% in 8–12 years group, and 16.3% in the 13 to 17 years age bracket. The most common tracer condition was eczema (35.3%), followed by asthma (26.4%) and constipation (15.8%). Twenty two percent had two or three conditions (multimorbidity). Both the index of multiple deprivation (IMD) and the Income Deprivation Affecting Children Index (IDACI) suggest that the majority of the study sample pertained to the most deprived quantiles, with 71% of individuals in the lower four IDACI quantiles. The majority of study participants had ethnic backgrounds other than White. The dimension and item score samples showed similar characteristics, except for a larger representation of the 8–12 years age group (accounting for 47.9%) and asthma prevalence (32.7%) in the item score sample. PedsQL scores ranged from 11.96 to 100, with a mean of 78.57 (SD = 17.82), while the CHU9D varied between 0.38 and 1, with a mean of 0.89 (SD = 0.11). The distributions of both variables were negatively skewed (Fig. 2). There was a positive correlation of 0.63 (p < 0.0001) between total PedsQL scores and CHU9D (Fig. 3). Maximum scores were observed in 6.78% and 17.63% of PedsQL and CHU9D responses, respectively.

**Table﻿ 1:** Sociodemographic and clinical characteristics of the study samples

	Full sample (*N* = 1225)		Dimension scores sample (*N* = 1198)		Item scores sample (*N* = 842)		Estimation sample (*N* = 674)		Validation sample (*N* = 168)	
Age categories, *n* (%)										
0–12 months	9	0.73%	–	–	–		–	–	–	–
13–24 months	18	1.47%	–	–	–		–	–	–	–
2–4 years	333	27.18%	333	27.80%	–		–	–	–	–
5–7 years	252	20.57%	252	21.04%	245	29.10%	204	30.27%	41	24.40%
8–12 years	413	33.71%	413	34.47%	404	47.98%	320	47.48%	84	50.00%
13–17 years	200	16.33%	200	16.69%	193	22.92%	150	22.26%	43	25.60%
Sex, *n* (%)										
Female	578	47.18%	569	47.50%	402	47.74%	320	47.48%	82	48.81%
Male	647	52.82%	629	52.50%	440	52.26%	354	52.52%	86	51.19%
Tracer condition, *n* (%)										
Asthma only	323	26.37%	322	26.88%	275	32.66%	217	32.20%	58	34.52%
Constipation only	194	15.84%	188	15.69%	95	11.28%	78	11.57%	17	10.12%
Eczema only	433	35.35%	415	34.64%	249	29.57%	207	30.71%	42	25%
Multimorbidity	275	22.45%	273	22.79%	223	26.48%	172	25.52%	51	30.36%
IMD (2019), *n* (%)										
1—Most deprived	23	1.88%	22	1.84%	13	1.54%	10	1.48%	3	1.79%
2	282	23.02%	276	23.04%	198	23.52%	162	24.04%	36	21.43%
3	361	29.47%	352	29.38%	244	28.98%	198	29.38%	46	27.38%
4	204	16.65%	200	16.69%	151	17.93%	122	18.10%	29	17.26%
5	131	10.69%	127	10.60%	87	10.33%	66	9.79%	21	12.50%
6	134	10.94%	132	11.02%	84	9.98%	70	10.39%	14	8.33%
7	45	3.67%	44	3.67%	33	3.92%	25	3.71%	8	4.76%
8	35	2.86%	35	2.92%	27	3.21%	18	2.67%	9	5.36%
9	10	0.82%	10	0.83%	5	0.59%	3	0.45%	2	1.19%
10—Least deprived	0	0.00%	0	0.00%	0	0.00%	0	0	0	0.00%
IDACI (2019), *n* (%)										
1—Most deprived	189	15.43%	184	15.36%	136	16.15%	111	16.47%	25	14.88%
2	380	31.02%	373	31.14%	273	32.42%	213	31.60%	60	35.71%
3	240	19.59%	234	19.53%	162	19.24%	132	19.58%	30	17.86%
4	129	10.53%	125	10.43%	84	9.98%	72	10.68%	12	7.14%
5	84	6.86%	81	6.76%	47	5.58%	35	5.19%	12	7.14%
6	70	5.71%	69	5.76%	47	5.58%	41	6.08%	6	3.57%
7	69	5.63%	68	5.68%	49	5.82%	40	5.93%	9	5.36%
8	40	3.27%	40	3.34%	27	3.21%	19	2.82%	8	4.76%
9	16	1.31%	16	1.34%	10	1.19%	7	1.04%	3	1.79%
10—Least deprived	8	0.65%	8	0.67%	7	0.83%	4	0.59%	3	1.79%
Ethnicity, *n* (%)										
White	445	36.33%	437	36.48%	287	34.09%	228	33.83%	59	35.12%
Black	381	31.10%	372	31.05%	288	34.20%	235	34.87%	53	31.55%
Mixed	218	17.80%	215	17.95%	146	17.34%	108	16.02%	38	22.62%
Asian	69	5.63%	65	5.43%	44	5.23%	39	5.79%	5	2.98%
Other ethnic groups	48	3.92%	48	4.01%	33	3.92%	30	4.45%	3	1.79%
Prefer not to say	41	3.35%	41	3.42%	30	3.56%	23	3.41%	7	4.17%
Missing	23	1.88%	20	1.67%	14	1.66%	11	1.63%	3	1.79%

White ethnicity includes White British, White Irish and White Any other White background. Black ethnicity includes Caribbean, African, and Any other Black background. Mixed ethnicity includes White and Black Caribbean, White and Black African, White and Asian, and any other Mixed background. Asian includes Indian, Pakistani, Bangladeshi, and Any other Asian background. Other ethnic groups include Chinese and Any other ethnic group

*IMD* Index of Multiple Deprivation, *IDACI* Income Deprivation Affecting Children Index

### External validation of published mapping algorithms

The goodness of fit of the three Sweeney mappings, applied to the CYPHP sample, is presented in Table 2. Information is displayed for each mapping sample (total, dimension, and item-level) and age and tracer condition subgroups. Overall, the Sweeney mappings perform well in the CYPHP sample compared with values reported in previous mappings, particularly the dimension equation. There is a slight underprediction in mean CHU9D scores across most groups, e.g. 0.88 versus 0.82 for the total scores mapping. However, for 2–4 years, 5–7 years, and 8–12 years age subgroups in the dimension score mapping, there was a small overprediction of the mean. All MAEs (lower values indicate better fit) are between 0.056 (5–7 years, dimension score) and 0.11 (multimorbidity, total score) and, thus, within the lower bound of previously published estimates, which ranged from 0.074 to 0.230 [19, 20]. The percentage of absolute errors smaller than 0.05 varies from 30% (multimorbidity, item score) to 61% (5–7 years, dimension score). Similar conclusions on the performance of the Sweeney algorithms across these two population subgroups are supported by the RMSE and the average error.

**Table 2:** External validation—goodness-of-fit results from total, dimension, and item-level mappings by age and health status

Group	*N*	Mean		Min. Max				Correlation coefficient	*R*^2^	MAE	MSE	RMSE	Absolute errors < 0.05 (%)	Average error
		Observed	Predicted	Observed		Predicted								
Panel A—Total score mapping														
Total	1225	0.888	0.823	0.384	1	0.331	0.992	0.625	0.391	0.092	0.016	0.107	40.49	0.065
0–12 months	9	0.902	0.881	0.857	1	0.783	0.976	0.140	0.020	0.069	0.006	0.082	33.33	0.021
13–24 months	18	0.922	0.861	0.710	1	0.658	0.975	0.783	0.613	0.064	0.008	0.067	61.11	0.061
2–4 years	333	0.912	0.878	0.581	1	0.393	0.992	0.593	0.352	0.069	0.009	0.088	49.55	0.034
5–7 years	252	0.892	0.811	0.507	1	0.429	0.992	0.337	0.581	0.102	0.019	0.111	36.51	0.081
8–12 years	413	0.878	0.796	0.384	1	0.380	0.992	0.618	0.382	0.105	0.020	0.114	35.59	0.082
13–17 years	200	0.861	0.798	0.481	1	0.331	0.992	0.661	0.437	0.095	0.016	0.109	39.00	0.063
Asthma only	323	0.880	0.827	0.464	1	0.396	0.992	0.605	0.366	0.085	0.014	0.105	42.11	0.052
Constipation only	194	0.873	0.810	0.504	1	0.380	0.992	0.701	0.492	0.093	0.015	0.105	37.63	0.063
Eczema only	433	0.911	0.852	0.467	1	0.393	0.992	0.564	0.318	0.086	0.014	0.105	45.27	0.059
Multimorbidity	275	0.872	0.783	0.384	1	0.331	0.992	0.627	0.394	0.111	0.021	0.112	33.09	0.090
Panel B—Dimension score mapping														
Total	1198	0.887	0.873	0.384	1	0.249	0.988	0.634	0.402	0.071	0.010	0.098	51.59	0.014
2–4 years	333	0.912	0.953	0.581	1	0.748	0.988	0.532	0.283	0.059	0.007	0.072	58.86	− 0.042
5–7 years	252	0.892	0.899	0.507	1	0.656	0.982	0.663	0.439	0.056	0.006	0.079	61.11	− 0.007
8–12 years	413	0.878	0.841	0.384	1	0.339	0.975	0.693	0.480	0.073	0.010	0.092	48.91	0.037
13–17 years	200	0.861	0.774	0.481	1	0.249	0.956	0.721	0.520	0.106	0.019	0.107	33.00	0.086
Asthma only	322	0.879	0.861	0.464	1	0.339	0.988	0.687	0.472	0.067	0.008	0.089	51.55	0.018
Constipation only	188	0.871	0.881	0.504	1	0.249	0.988	0.671	0.450	0.070	0.010	0.099	52.66	− 0.010
Eczema only	415	0.911	0.905	0.467	1	0.433	0.988	0.535	0.286	0.065	0.008	0.092	57.59	0.007
Multimorbidity	273	0.872	0.835	0.384	1	0.339	0.987	0.626	0.392	0.085	0.013	0.110	41.76	0.037
Panel C—Item-level mapping														
Total	842	0.877	0.809	0.384	1	0.372	1	0.616	0.379	0.098	0.017	0.112	37.05	0.069
5–7 years	245	0.891	0.813	0.507	1	0.410	1	0.535	0.286	0.104	0.020	0.119	37.96	0.078
8–12 years	404	0.878	0.807	0.384	1	0.434	1	0.642	0.412	0.098	0.017	0.110	35.64	0.071
13–17 years	193	0.860	0.807	0.481	1	0.372	1	0.660	0.436	0.089	0.014	0.107	38.86	0.052
Asthma only	275	0.874	0.832	0.464	1	0.464	1	0.590	0.348	0.084	0.013	0.108	42.55	0.043
Constipation only	95	0.844	0.777	0.504	1	0.380	1	0.731	0.535	0.097	0.015	0.103	31.58	0.066
Eczema only	249	0.907	0.827	0.467	1	0.481	1	0.533	0.284	0.101	0.020	0.116	38.96	0.080
Multimorbidity	223	0.863	0.774	0.384	1	0.372	1	0.652	0.425	0.111	0.021	0.113	30.49	0.089

### Developing new mapping algorithms

The variables selected based on the estimation sample and the AIC criteria for the total, dimension and item-level equations are listed in the first column of Table 3. The same variables were included in the model specification for the total and dimension-level equations when forward selection was used; the item-level specification differed slightly but yielded similar goodness of fit to the AIC approach (Table S1).

**Table 3:** Best mapping equations from PedsQL to CHU9D utility scores

Variable	Total score model			Dimension score model			Item model		
	Parameter	SE	*p*	Parameter	SE	*p*	Parameter	SE	*p*
Constant	0.58679	0.04551	< .0001	0.58625	0.04064	< .0001	0.50166	0.03533	< .0001
Age	− 0.00272	0.00115	0.0178	− 0.00232	0.00105	0.0281	− 0.00160	0.00111	0.1516
PedsQL total	0.00484	0.00130	0.0002						
PedsQL total^2^	− 0.00001	0.00001	0.4056						
Physical dimension				− 0.00150	0.00106	0.1589			
Emotional dimension				0.00570	0.00073	< .0001			
School dimension				0.00106	0.00022	< .0001			
Social dimension				0.00012	0.00104	0.9115			
Physical dimension^2^				0.00002	0.00001	0.0404			
Emotional dimension^2^				− 0.00002	0.00001	< .0001			
Social dimension^2^				0.00000	0.00001	0.0281			
Item 3							− 0.00028	0.00048	0.5635
Item 4							− 0.00023	0.00015	0.1355
Item 5							− 0.00021	0.00013	0.1009
Item 6							− 0.00008	0.00046	0.8585
Item 7							0.00163	0.00050	0.0012
Item 8							0.00067	0.00016	< .0001
Item 9							0.00213	0.00064	0.0009
Item 10							− 0.00027	0.00078	0.7265
Item 11							0.00157	0.00054	0.0037
Item 12							0.00111	0.00052	0.0329
Item 13							− 0.00087	0.00060	0.1452
Item 14							− 0.00088	0.00051	0.0822
Item 15							0.00149	0.00074	0.0456
Item 17							− 0.00084	0.00061	0.1662
Item 19							− 0.00090	0.00047	0.0546
Item 20							0.00165	0.00056	0.0031
Item 21							− 0.00015	0.00048	0.7557
Item 23							0.00186	0.00067	0.0061
Item 3^2^							0.000003	0.00000	0.3660
Item 6^2^							0.000003	0.00000	0.3876
Item 7^2^							− 0.00001	0.00000	0.0047
Item 9^2^							− 0.00001	0.00000	0.0057
Item 10^2^							0.000004	0.00001	0.4870
Item 11^2^							− 0.00001	0.00000	0.0460
Item 12^2^							− 0.000005	0.00000	0.2321
Item 13^2^							0.00001	0.00000	0.0788
Item 14^2^							0.00001	0.00000	0.2117
Item 15^2^							− 0.00001	0.00001	0.0782
Item 17^2^							0.00001	0.00000	0.1240
Item 19^2^							0.00001	0.00000	0.0518
Item 20^2^							− 0.00001	0.00000	0.0109
Item 21^2^							0.000004	0.00000	0.2700
Item 23^2^							− 0.00001	0.00000	0.0485

Variables selected based on AIC criterion and models estimated using OLS. The estimation sample was used (*N* = 674). See Table S2 for a listing of the PedsQL items included in the CYPHP mappings. To use these mappings, parameter estimates should be multiplied by the observed demographic and PedsQL score values. For example, the CHU9D value for a 10-year-old individual, with a total PedsQL score of 75 would be equal to 0.58679 + (− 0.00272 * 10) + (0.00484 * 75) + (− 0.00001 * 75^2^) = 0.866

The best estimation method for the three final equations was OLS, followed by CLAD, GLM, and BETA (Table 4). OLS performs better across the majority of goodness of fit measures compared to the other estimation methods, providing a particularly precise mean prediction matching observed values (Fig. S1).

**Table 4:** Prediction summary of estimation sample—goodness-of-fit results from total, dimension, and item-level mappings

Group	*N*	Mean	Min	Max	Correlation coefficient	*R*^2^	MAE	MSE	RMSE	Absolute errors < 0.05 (%)	Average error
Panel A—Total score mapping											
Observed CHU9D	674	0.879	0.384	1	–	–	–	–	–	–	–
OLS	674	0.879	0.607	0.980	0.624	0.390	0.066	0.008	0.089	51.63	0
GLM	674	0.879	0.616	0.981	0.623	0.388	0.066	0.008	0.089	51.63	0
BETA	674	0.886	0.746	0.975	0.580	0.336	0.068	0.009	0.092	52.23	− 0.007
CLAD	674	0.890	0.573	0.997	0.624	0.389	0.064	0.008	0.089	53.12	− 0.012
Panel B—Dimension score mapping											
Observed CHU9D	674	0.879	0.384	1	–	–	–	–	–	–	–
OLS	674	0.879	0.537	0.990	0.712	0.506	0.059	0.006	0.080	54.45	0
GLM	674	0.879	0.577	0.991	0.708	0.502	0.059	0.006	0.080	53.41	0
BETA	674	0.886	0.480	0.980	0.676	0.458	0.062	0.007	0.084	55.93	− 0.007
CLAD	674	0.889	0.515	1	0.711	0.505	0.058	0.007	0.080	56.38	− 0.010
Panel C—Item-level mapping											
Observed CHU9D	648	0.878	0.384	1	–	–	–	–	–	–	–
OLS	648	0.878	0.442	1	0.761	0.579	0.055	0.005	0.074	59.79	0
GLM	648	0.878	0.489	1*	0.760	0.578	0.055	0.005	0.074	59.2	0
BETA	648	0.884	0.418	0.986	0.715	0.511	0.059	0.007	0.081	60.24	− 0.007
CLAD	648	0.889	0.453	1	0.749	0.561	0.053	0.006	0.075	62.91	− 0.011

The sample size for the item-level mapping is slightly smaller (648 vs. 674) due to missing values in some items

*CHU9D predicted values were truncated at 1

The twelve models with the AIC-based specifications were applied to the validation sample and goodness of fit further assessed (Table 5). The total, dimension, and item-level models display an acceptable goodness of fit, with MAE values around 0.06. The final models display an accurate prediction of the mean CHU9D, with only a slight overprediction (e.g., for the total score mapping, 0.881 versus 0.872). All VIF values were below 3.5 suggesting multicollinearity is not an issue (Table S3).

**Table 5:** Prediction summary of validation sample—goodness-of-fit results from total, dimension, and item-level mappings

Group	*N*	Mean	Min	Max	Correlation coefficient	*R*^2^	MAE	MSE	RMSE	Absolute errors < 0.05 (%)	Average error
Panel A—Total score mapping											
Observed CHU9D	168	0.872	0.464	1	–	–	–	–	–	–	–
OLS	168	0.881	0.648	0.972	0.664	0.440	0.066	0.008	0.088	47.62	− 0.009
GLM	168	0.881	0.651	0.972	0.664	0.441	0.066	0.008	0.088	47.62	− 0.009
BETA	168	0.887	0.749	0.971	0.625	0.390	0.067	0.009	0.092	48.81	− 0.015
CLAD	168	0.893	0.619	0.990	0.661	0.437	0.065	0.008	0.087	54.17	− 0.021
Sweeney	168	0.8061	0.379	0.991	0.651	0.424	0.066	0.015	0.106	37.5	0.066
Panel B—Dimension score mapping											
Observed CHU9D	168	0.872	0.464	1	–	–	–	–	–	–	–
OLS	168	0.880	0.683	0.982	0.684	0.469	0.064	0.007	0.085	51.19	− 0.009
GLM	168	0.880	0.678	0.982	0.684	0.468	0.064	0.007	0.085	51.79	− 0.008
BETA	168	0.885	0.657	0.977	0.643	0.414	0.064	0.008	0.089	56.55	− 0.013
CLAD	168	0.890	0.669	0.999	0.681	0.464	0.064	0.008	0.085	55.36	− 0.018
Sweeney	168	0.841	0.420	0.978	0.652	0.426	0.072	0.010	0.098	50	0.031
Panel C—Item-level mapping											
Observed CHU9D	161	0.873	0.464	1	–	–	–	–	–	–	–
OLS	161	0.882	0.633	0.984	0.697	0.486	0.062	0.007	0.084	55.95	− 0.009
GLM	161	0.882	0.639	0.993	0.688	0.474	0.063	0.007	0.085	57.14	− 0.009
BETA	161	0.889	0.588	0.979	0.689	0.474	0.063	0.007	0.085	58.93	− 0.016
CLAD	161	0.894	0.554	1	0.718	0.515	0.060	0.007	0.082	61.9	− 0.020
Sweeney	161	0.812	0.410	1	0.638	0.407	0.060	0.016	0.110	34.52	0.060

The sample size for the item-level mapping is slightly smaller (161 vs. 168) due to missing values in some items

The parameter estimates of the final mapping equations based on the CYPHP sample are presented in Table 3. Compared with previous mappings, our equations are characterised by a higher intercept, the presence of age (rather than sex) and more non-linear terms to describe the relationship between the PedsQL and the CHU9D. These new mappings perform better for the CYPHP sample compared to previously published mappings. For example, for the total score mapping, using Sweeney et al. existing mappings provided an estimated mean of 0.82 compared to an observed mean of 0.89. The MAE was 0.092, the RMSE 0.1071, and the percentage of absolute errors < 0.05 was 40.49% (Table 2). The corresponding values for the CYPHP mapping (using the OLS algorithm) are an estimated mean of 0.88 vs. observed mean of 0.87, a MAE of 0.066, a RMSE of 0.088, and the percentage of absolute errors < 0.05 of 47.62% (Table 5). The new mapping algorithms, particularly using OLS, outperformed the Sweeney mapping in the validation sample (Table 5). Within the CYPHP mappings, the OLS item-level equation yields the most accurate prediction of CHU9D scores.

The CYPHP mappings for dimension and total scores are positively related to the Sweeney mappings (Table S4). The results of the regression analysis confirm our previous results that, on average, the predictive scores using the CYPHP models are higher than those estimated using the Sweeney models. The exclusion of demographic characteristics (age in this case) resulted in little to no change to the model performance (Table S5).

## Discussion

This is the first paper to externally validate the most recent PedsQL to CHU9D mappings using a sample of children and young people with chronic conditions from an ethnically diverse and deprived area in South London. After analysing these results, we determined whether improvements to the equations could be made for this diverse population. Our results indicate that, while existing mappings have an acceptable performance in the CYPHP sample, even better mappings can be built. The new CYPHP algorithms for mapping PedsQL onto CHU9D, particularly using OLS, yield superior goodness of fit compared previously published mappings.

The new mappings are characterised by the presence of age as an important predictor, and more non-linear terms compared to previous work. Future research should assess the potential improvement to goodness of fit measures by adding variables such as socioeconomic status or medical information to model specifications. The importance of age, rather than sex, in the CYPHP mappings could be explained by the wider age range covered by the CYPHP sample (0–16 years) compared to previous mappings (e.g., 10–12 years in the Sweeney et al. mapping [19]). The sex split of the CYPHP sample is similar to previous papers, however inclusion of sex as a variable did not significantly improve the predictive accuracy of our models. The presence of more squared terms in the CYPHP equations underscores the nuanced relationship between the PedsQL and the CHU9D, and the challenges entailed in linking a measure with 23 items (PedsQL) to one with 9 items (CHU9D), particularly for a developing, paediatric population. Both instruments cover physical, emotional, social and school functioning, yet the wording of the questions and their depth are different. This points to the conceptual limitations of mapping algorithms and the preference for direct data collection when possible.

Direct measurement of HRQoL for clinical use or for research purposes (e.g. evaluation and cost effectiveness analysis) may not always be possible. The ability to predict QALYs when only PedsQL scores and demographic characteristics are available becomes particularly useful. For example, this could allow the accurate prediction of missing values when a CHU9D response is not available. It could perhaps reduce the cost and time burden of filling out two separate HRQoL instruments, when one would suffice. Yet it is important to highlight the need to account for the uncertainty around the predicted CHU9D values. A caveat to the current study findings is that, although a population of children and young people across a wide age range was included, direct measurement, rather than use of mapping algorithms, may be particularly desirable among children below 5 years of age. This group was excluded from the item-level mapping as PedsQL items were considered too heterogenous to be pooled with the rest of the sample. Additionally, children under two years of age were removed from the dimension-level mapping as physical symptoms and cognitive function dimensions are not measured in older age groups, while school function is not relevant yet among the younger group. A strength of both HRQoL instruments is that they are self-completed. Children of a certain age or condition may require an adult or guardian to complete the questions introducing proxy biases. By completing two different HRQoL instruments, it may be possible to identify or reduce bias. The majority of the current population (77.4%) had one chronic condition only.

Although the performance of mapping algorithms was acceptable for the multimorbid population, the evidence on the accuracy of existing and new mappings for this subpopulation is limited. As suggested by Sweeney et al., further research is required to determine if direct measurement compared to mapping algorithms are more appropriate to use for children with lower HRQoL. Additionally, further validations of the CYPHP mappings for children and young people with multimorbidity or with mental health conditions is needed.

One of the strengths of this study is that it is the first to externally validate existing mappings with a sample of children and young people under 16 years of age with chronic conditions in a UK deprivated urban area. Previous mappings were based on an Australian sample with limited age coverage (15–17 years [18, 24] and 10–12 years of age [19]) or children in the UK with corticosteroid-sensitive nephrotic syndrome [20]. This paper also developed PedsQL to CHU9D mapping equations with enhanced predictive accuracy. By conducting external and internal validations using a wider age range, this paper aims to contribute to NICE’s recommendation on choosing HRQoL instruments with good psychometric performance in the relevant age range. The new equations are aimed at complementing existing ones so that researchers seeking CHU9D scores from PedsQL questionnaires can select the mapping algorithm most appropriate for their study population.

Study limitations included a relatively low representation of children below 2 years of age, which requires further examination of existing and new mappings among this younger population. Second, the majority of our sample (77.4%) had one chronic condition only. Even though the performance of mapping algorithms was acceptable for the multimorbid population, the evidence on the accuracy of existing and new mappings for this subpopulation is limited.

## Conclusions

This study presents mapping algorithms that can predict CHU9D scores from PedsQL scores with good accuracy. Additionally, these new algorithms out performed previous algorithms that were also externally validated in this paper. The ability to predict CHU9D scores when only PedsQL scores and demographics are available is useful for economic evaluation and meta-analysis. The new CYPHP mappings are particularly relevant for samples with children and young people with chronic conditions living in deprived and urban settings, however, further validation in an external sample is required.

## Supplementary Information

Below is the link to the electronic supplementary material.

Supplementary file1 (DOCX 292 KB)

## Data Availability

The data that support the findings of this study are available from the corresponding author on reasonable request.
