# Supplementary material for: Mapping PedsQL™ scores to CHU9D utility weights for children with chronic conditions in a multi-ethnic and deprived metropolitan population
Source: Qual Life Res. 2023 Feb 23;32(7):1909–23. doi: 10.1007/s11136-023-03359-4 (PMC10241699; doi:10.1007/s11136-023-03359-4)
Supplement: Supplementary file 1 — Supplementary file1 (DOCX 292 KB) [file 11136_2023_3359_MOESM1_ESM.docx]

**Supplement**

| **Table S1. Variable selection based on forward selection--goodness-of-fit results from item-level mapping in estimation and validation samples** | | | | | | | | | | | | | | | | | | | | |
| --- | --- | --- | --- | --- | --- | --- | --- | --- | --- | --- | --- | --- | --- | --- | --- | --- | --- | --- | --- | --- |
|  | |  | | |  | |  | |  | |  | | |  | |  | |  |  | |
| **Group** | | **N** | **Mean** | | **Min, Max** | | | | **Correlation coefficient** | | **R-squared** | **MAE** | | **MSE** | | **Absolute errors<0.05** | | |  |  |
| **Estimation sample** | |  |  | |  | |  | |  | |  |  | |  | |  | | |  |  |
| Observed CHU9D | | 674 | 0.879 | | 0.384 | | 1 | | - | | - | - | | - | | - | | |  |  |
| OLS -AIC | | 674 | 0.879 | | 0.607 | | 0.980 | | 0.62416 | | 0.38958 | 0.06584 | | 0.00781 | | 51.63 | | |  |  |
| OLS -forward selection | | 674 | 0.879 | | 0.630 | | 0.984 | | 0.62365 | | 0.38894 | 0.06592 | | 0.00782 | | 51.78 | | |  |  |
| **Validation sample** | |  |  | |  | |  | |  | |  |  | |  | |  | | |  |  |
| Observed CHU9D | | 168 | 0.872 | | 0.464 | | 1 | | - | | - | - | | - | | - | | |  |  |
| OLS -AIC | | 168 | 0.881 | | 0.648 | | 0.972 | | 0.66368 | | 0.44047 | 0.06555 | | 0.00772 | | 47.62 | | |  |  |
| OLS - forward selection | | 168 | 0.881 | | 0.666 | | 0.976 | | 0.66308 | | 0.43968 | 0.06553 | | 0.00773 | | 48.21 | | |  |  |

Notes: For the total and dimension-level models, forward selection resulted in the same selected variables as the AIC-based criterion.

**Table S2. PedsQL items in CYPHP mapping**

|  | **PedsQL TM 4.0 items** |
| --- | --- |
| 3 | Participating in sports activity or exercise |
| 4 | Lifting something heavy |
| 5 | Taking a bath or shower by him or herself |
| 6 | Doing chores, like picking up his or her toys/around the house |
| 7 | Having hurts or aches |
| 8 | Low energy level |
| 9 | Feeling afraid or scared |
| 10 | Feeling sad or blue |
| 11 | Feeling angry |
| 12 | Trouble sleeping |
| 13 | Worrying about what will happen to him or her |
| 14 | Getting along with other children/teens |
| 15 | Other kids/teens not wanting to be his or her friend |
| 17 | Not able to do things that other children/teens his or her age can do |
| 19 | Paying attention in class |
| 20 | Forgetting things |
| 21 | Keeping up with school activities |
| 23 | Missing school to go to doctor or hospital |

**Table S3. Assessment of multicollinearity among regressors of the CYPHP mappings**

| **Regressor** | **VIF** |
| --- | --- |
| **Dimension mapping** | |
| **Physical** | 2.08 |
| **Emotional** | 1.66 |
| **School** | 2.53 |
| **Social** | 2.60 |
| **Age** | 1.03 |
| **Item mapping** | |
| **I3** | 1.83 |
| **I4** | 1.63 |
| **I5** | 1.90 |
| **I6** | 2.07 |
| **I7** | 1.74 |
| **I8** | 1.89 |
| **I9** | 2.42 |
| **I10** | 2.48 |
| **I11** | 1.91 |
| **I12** | 1.78 |
| **I13** | 2.40 |
| **I14** | 2.21 |
| **I15** | 2.04 |
| **I17** | 1.76 |
| **I19** | 3.04 |
| **I20** | 1.94 |
| **I21** | 3.34 |
| **I23** | 1.50 |
| **Age** | 1.17 |

**Table S4. Parameter estimates from regressions assessing the relationship between the CYPHP and Sweeney mappings**

| **1. Total scores** |  |  |  |  |
| --- | --- | --- | --- | --- |
| **Parameter** | **Estimate** | **Standard Error** | **t Value** | **Pr > \|t\|** |
| **Intercept** | 0.4800 | 0.0046 | 104.77 | <.0001 |
| **CHU9D_Sweeney_Total** | 0.4972 | 0.0056 | 88.67 | <.0001 |
|  |  |  |  |  |
| **2. Dimension scores** |  |  |  |  |
| **Parameter** | **Estimate** | **Standard Error** | **t Value** | **Pr > \|t\|** |
| **Intercept** | 0.4159 | 0.0177 | 23.55 | <.0001 |
| **CHU9D_Sweeney_Dimension** | 0.5521 | 0.0208 | 26.55 | <.0001 |

Notes: Both regression models were estimated in the validation dataset (N=167) and had as a dependent variable the CHU-9D score resulting from the best CYPHP mapping and the Sweeney CHU-9D score as the independent variable.

**Figure S1. Predicted versus observed values for the final CYPHP mappings**

1. **Total scores**


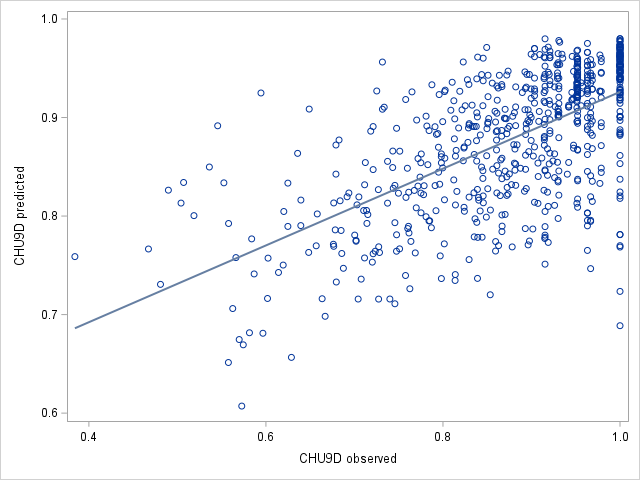


1. **Dimension scores**


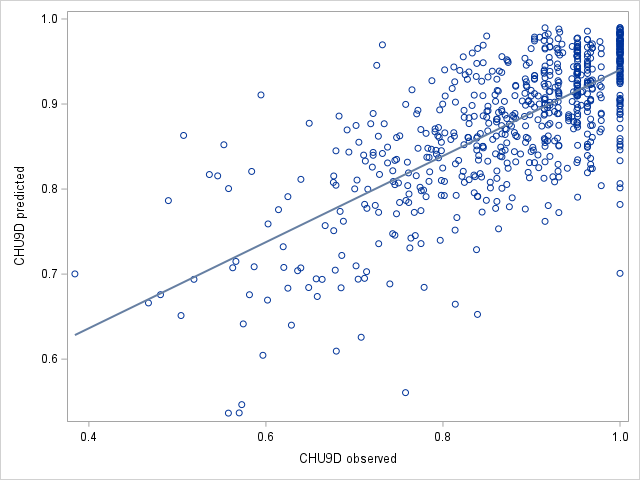


1. **Item scores**


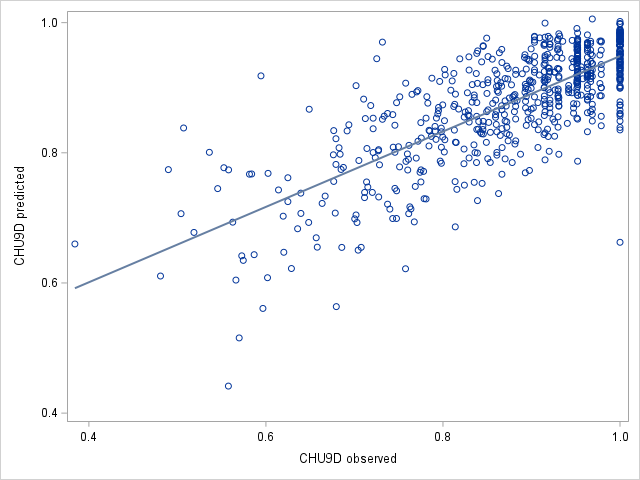


Notes: All three graphs are based on predicted and observed values from the estimation sample (N=674).

**Table S5. Comparison of goodness of fit measures when age is dropped from the CYPHP dimension mapping**

| **Group** | **N** | **Mean** | **Min, Max** | | **Correlation coefficient** | **R-squared** | **MAE** | **MSE** | **RMSE** | **Absolute errors<0.05** | **Average error** |
| --- | --- | --- | --- | --- | --- | --- | --- | --- | --- | --- | --- |
| Estimation dataset | | | | | | | | | | |  |
| Observed CHU9D | 674 | 0.879 | 0.384 | 1 | - | - | - | - | - | - | - |
| OLS | 674 | 0.879 | 0.537 | 0.99 | 0.712 | 0.506 | 0.0589 | 0.0063 | 0.0795 | 54.45% | 0 |
| OLS without age | 674 | 0.879 | 0.532 | 0.98 | 0.709 | 0.503 | 0.0591 | 0.0064 | 0.0798 | 54.6% | 0 |
| Validation dataset | | | | | | | | | | |  |
| Observed CHU9D | 168 | 0.872 | 0.464 | 1 | - | - | - | - | - | - | - |
| OLS | 168 | 0.88 | 0.683 | 0.98 | 0.684 | 0.4685 | 0.0635 | 0.0073 | 0.085 | 51.19% | -0.008 |
| OLS without age | 168 | 0.88 | 0.67 | 0.978 | 0.672 | 0.4517 | 0.064 | 0.0075 | 0.087 | 53.57% | -0.009 |
